# Ultrasonographic Characteristics of Thyroid Nodules with Nondiagnostic and Atypia of Undetermined Significance in Fine-Needle Aspiration Cytology

**DOI:** 10.5334/jbsr.3577

**Published:** 2024-05-07

**Authors:** Ahmet Bozer, Hülya Çetin Tunçez, Tuğçe Doğa Kul, Asuman Argon

**Affiliations:** 1 Department of Radiology, İzmir City Hospital, İzmir, Turkey; 2 Department of Radiology, İzmir City Hospital, İzmir, Turkey; 3 Department of Radiology, İzmir City Hospital, İzmir, Turkey; 4 Department of Pathology, İzmir City Hospital, İzmir, Turkey

**Keywords:** Thyroid nodule, Fine Needle Aspiration Cytology (FNAC), nondiagnostic, atypia of undetermined significance, ultrasound

## Abstract

**Objectives:**

This study aimed to investigate ultrasound (US) features of thyroid nodules categorized as nondiagnostic (ND) and atypia of undetermined significance (AUS) according to the Bethesda System for Reporting Thyroid Cytopathology (TBSRTC) and their potential implications for clinical management.

**Materials and Methods:**

A retrospective study was conducted on patients who underwent thyroid nodules FNAC between 2019 and 2023. Nodules falling into the ND and AUS categories were analyzed for US features, nodule size, composition, echogenicity, shape, margin, echogenic foci, the distribution of the American College of Radiology’s Thyroid Imaging Reporting and Data System (ACR TI-RADS) categories, and other parameters. The study included a total of 1,199 patients and 1,252 nodules (ND: 1110; AUS: 142).

**Results:**

No significant differences in age, gender, nodule features, echogenicity, shape, margin, echogenic foci, TI-RADS scores, localization, number of nodules, or thyroid parenchymal disease presence were found between the ND and AUS categories (*p* > 0.05). Also, no statistically significant difference in nodule size (<10 mm vs. ≥10 mm) existed between the ND and AUS categories (*p* = 0.475). Both showed predominantly solid composition and hyperechoic/isoechoic echogenicity. High proportions of TI-RADS 4 nodules were observed in both groups, with 727 (65.5%) in ND and 95 (66.9%) in AUS.

**Conclusion:**

This study found no statistically significant differences in US characteristics between the ND and AUS categories, indicating potential similarities in their radiological appearances. Also, no significant difference in nodule size (<10 mm and ≥10 mm) was observed between these categories. Clinical management should consider further investigations, including repeat FNAC, due to the diagnostic challenges and malignancy risk in both categories.

## Introduction

Thyroid nodules, among the most common lesions affecting the thyroid gland, encompass both benign and malignant conditions, constituting a significant portion of clinical practice [1]. The diagnosis and management of thyroid nodules can profoundly impact patients’ treatment options and outcomes. Fine needle aspiration cytology (FNAC) is a significant and effective diagnostic tool for evaluating thyroid nodules [2]. The Bethesda System for Reporting Thyroid Cytopathology (TBSRTC) is a standardized classification used by pathologists and clinicians to interpret FNAC results for thyroid nodules. It categorizes findings from benign to potentially malignant, providing a common language for healthcare professionals. This standardization enhances communication, reduces ambiguity, and aids in making informed decisions about further evaluation and treatment for patients with thyroid nodules [3].

The TBSRTC classifies thyroid nodule FNAC results into six categories, ranging from nondiagnostic (ND) material for diagnosis to confirmed malignancy. The malignancy rates for each category are as follows: Category I (ND) (13%), Category II (Benign) (4%), Category III (AUS—Atypia of Undetermined Significance) (22%), Category IV (FN—Follicular Neoplasm) (30%), Category V (SFM – Suspicious for Malignancy) (74%), and Category VI (Malignant) (97%). In its 2023 edition, TBSRTC resolved confusion by recommending a single designation for each of the six diagnostic categories, discontinuing alternative names such as ‘unsatisfactory,’ ‘follicular lesion of undetermined significance,’ and ‘suspicious for a follicular neoplasm’ [4].

The diagnostic challenges posed by thyroid nodules, specifically within the AUS or ND categories according to TBSRTC, require attention due to their significant clinical impact. These categories neither confirm benignity nor establish malignancy; however, they carry a malignancy risk ranging from 1% to 15%, leaving patients in diagnostic uncertainty. Alongside interinstitutional disparities, variable malignancy rates have been demonstrated in nodules categorized as AUS, ranging from 33% to 46% [5–7].

While many studies have examined ultrasound (US) features in thyroid nodules with suspicious cytology to differentiate benign from malignant ones, research on nodules categorized as AUS and ND is limited. Despite TBSRTC improving FNAC interpretation, further investigation is needed for these categories. This study specifically analyzes US features of nodules in these categories, aiming to identify markers to enhance diagnostic accuracy. By assessing these characteristics, our goal is to offer insights for clinical decision-making, potentially reducing invasive procedures and optimizing thyroid nodule management.

## Materials and Methods

### Participant Selection

This retrospective study, ethically approved by the Institutional Review Board (Decision No.: 2023/87), encompassed patients who underwent FNAC for thyroid nodules at our hospital between 2019 and 2023. Inclusion criteria required patients to have a diagnosis of thyroid nodules falling under categories I (ND) or III (AUS), while exclusion criteria encompassed patients with a prior history of thyroid cancer, prior thyroid surgeries or interventions, those lacking critical information necessary for analysis, and individuals under the age of 18.

The number of thyroid FNACs performed within the specified date range was 3,835. Among these, 1218 (32%) were in Category I, and 242 (6%) were in Category III. Twenty-four individuals under the age of 18, 50 patients with a history of prior surgeries, and 134 patients with incomplete information were excluded from the study. This resulted in the inclusion of a total of 1252 nodules and 1199 patients in the study.

The nodule that repeated FNAC from the same nodule and yielded the same result was included once. Nodules with repeated FNAC and two outcomes were included in both groups. In ND, there were repeated identical results in 341 nodules, while in AUS, there were repeated identical results in five nodules. These nodules were counted as single instances in the analysis.

### Ultrasonographic Evaluation

**Figure 1:**
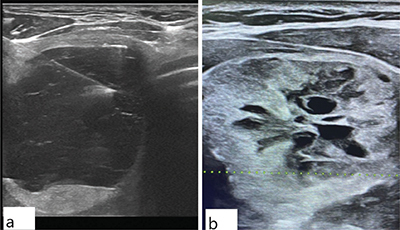
Ultrasound images of thyroid nodules with different categorizations. **(a)** This image depicts a nodule with cystic features, characterized by smooth margins and floating colloid, along with echogenic foci and debris (TI-RADS 1). The fine-needle aspiration cytology (FNAC) results for this nodule indicated a nondiagnostic (ND) outcome. **(b)** It exhibits a solid composition, an iso-hypoechoic appearance, a heterogeneous texture, central cystic regions, and a lobulated margin (TI-RADS 4). The FNAC results for this nodule indicated atypia of undetermined significance (AUS).

The assessment utilized 5 to 12 MHz linear array transducers in conjunction with the Samsung RS85 Ultrasound System (Samsung, Germany), adhering to the criteria specified in the American College of Radiology’s Thyroid Imaging Reporting and Data System (TI-RADS) (Figure 1) [8]. In addition to TI-RADS, several parameters associated with thyroid nodules were scrutinized, encompassing nodule size, location (right, left, isthmus), internal structure, number of nodules (solitary, multiple), and sonographic evidence of thyroid parenchymal disease (present, absent). The TI-RADS score for the nodules was calculated, and the corresponding categories were determined. The individual responsible for both performing the FNA procedure and evaluating the US features remained the same. Preceding the FNA, US characteristics were meticulously documented through a structured report.

### FNA Biopsy Technique

In adherence to ethical guidelines, informed consent was obtained from patients before the biopsy. Skin sterilization was achieved using a 10% povidone-iodine solution to ensure aseptic conditions. Local anesthesia was not administered. Nodules were selected based on TI-RADS criteria, and US-guided FNA biopsies, with a minimum of three passes per nodule for procedural precision, were performed using a 22 G needle and a disposable 10 mL syringe (Figure 1).

### Cytological Evaluation

**Figure 2:**
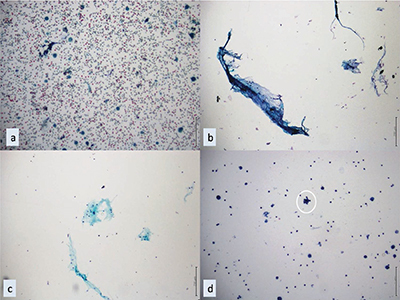
Cytopathological illustrations of nodules diagnosed as ND by FNAC. **(a)** A case of ND without follicular cells, where macrophages were observed in the background of bleeding (PAP, x10). **(b-c)** ND cases with only colloid and inflammatory cells observed in liquid-based cytology preparations (x10). **(d)** An ND case in which a single group of follicular cells was observed in addition to macrophages on the colloidal background; the thyrocyte group is highlighted in the circle (liquid-based cytology, x10). PAP: Papanicolaou Stain, ND: Nondiagnostic, FNAC: Fine Needle Aspiration Cytology.

**Figure 3:**
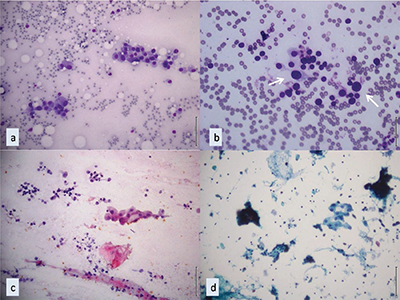
Cytopathological illustrations of nodules diagnosed as AUS by FNAC. **(a)** An AUS case displaying a small number of oncocytic follicular cells in non-colloidal hypocellular smears (MGG, x10). **(b)** An AUS case featuring a small number of follicular cells with nuclear enlargement and membrane irregularities in the predominantly benign preparation; atypical cells are indicated with white arrows (MGG, x20). **(c-d)** Atypical cyst-lining epithelial cells observed in the nodule, radiologically defined as cystic (c: PAP, magnification x10; d: liquid-based cytology, PAP, x10). MGG: May-Grünwald Giemsa, PAP: Papanicolaou Stain, AUS: Atypia of Undetermined Significance, FNAC: Fine Needle Aspiration Cytology.

The samples taken from the patient were spread on two thin smear slides. One of these slides was air-dried and stained with May Grunwald Giemza, while the other was fixed with alcohol and stained with Papanicolaou Stain (PAP). A PAP-stained preparation was prepared from the samples in red solution using liquid-based cytology (The BD Diagnostics, SurePath test), and a cell block was also created. The section obtained from the cell blocks was stained with hematoxylin and eosin. All preparations were evaluated by an experienced pathologist according to TBSRTC (Figures 2, 3).

### Statistical Evaluation

The data obtained in the study were entered into a database created in the Statistical Package for Social Sciences 26 (SPSS) program, and the statistical analysis of the data was performed using the SPSS package programs. Continuous variables were tested for their suitability for normal distribution, and it was determined that not all variables were compatible with normal distribution conditions. Quantitative variables were presented with median and interquartile range (IQR) values, and non-parametric methods were used for comparing these variables. Comparisons between independent groups were conducted using the Mann–Whitney U test. Qualitative variables were presented as frequencies and percentages in cross tables, and their distributions were compared using the Chi-Square or Fisher’s exact tests. In all tests, the significance level α was set at 0.05, and two-tailed tests were performed. If the *p*-value was less than 0.05, the difference between the groups was considered statistically significant.

## Results

The study examined a total of 1,252 nodules, with 1,100 nodules (87.8%) in Category I and 142 nodules (11.2%) in Category III (Table 1). The median age was 55 years, with an IQR of 46 to 65, and the gender distribution was 319 males (25.5%) and 933 females (74.5%). The median nodule size was found to be 15 mm, with an IQR of 12 to 22 mm. In Category I, the median nodule size was 15 (IQR: 11–22), and in Category III, it was 16 (IQR: 12–24). In both categories, the median TI-RADS score was 4 (IQR: 4–5).

**Table 1:** Comparison of characteristics of nodules in Bethesda Category I (ND) and Category III (AUS) based on FNA results.

		TOTAL *N* = 1152	CATEGORY I (ND) *N*_1_ = 1,110	CATEGORY III (AUS) *N*_2_ = 142	*P* (2-SIDED)
**Age** (Median, IQR)		55 (46–65)	55 (46–65)	54 (43–63)	*0.284*
**Nodule size (mm)** (Median, IQR)		15 (12–22)	15 (11–22)	16 (12–24)	*0.321*
**Nodule size (mm)** **(n, %)**	<10 mm	108 (8.6)	98 (8.8%)	10 (7.0%)	*0.475*
	≥10 mm	1144 (91.4)	1012 (91.2)	132 (93.0%)	
**TI-RADS Score** (Median, IQR)		4 (4–5)	4 (4–5)	4 (4–5)	*0.599*
**Gender** **(n, %)**	Male	319 (25.5%)	289 (26.0%)	30 (21.1%)	*0.206*
	Female	933 (74.5%)	821 (74.0%)	112 (78.9%)	
**Composition** **(n, %)**	Cystic or almost completely cystic	43 (3.4%)	37 (3.3%)	6 (4.2%)	*0.693*
	Spongiform	8 (0.6%)	8 (0.7%)	0 (0.0%)	
	Mixed cystic and solid	259 (20.7%)	228 (20.5%)	31 (21.8%)	
	Solid or almost completely solid	942 (75.2%)	837 (75.4%)	105 (73.9%)	
**Echogenicity** **(n, %)**	Anechoic	47 (3.8%)	41 (3.7%)	6 (4.2%)	*0.160*
	Hyper- or isoechoic	799 (63.8%)	699 (63.0%)	100 (70.4%)	
	Hypoechoic	406 (32.4%)	370 (33.3%)	36 (25.4%)	
**Shape** **(n, %)**	Wider-than-tall	1,242 (99.2%)	1,101 (99.2%)	141 (99.3%)	*1.000*
	Taller-than-wide	10 (0.8%)	9 (0.8%)	1 (0.7%)	
**Margin** **(n, %)**	Smooth—ill-defined	1,147 (91.6%)	1,020 (91.9%)	127 (89.4%)	*0.585*
	Lobulated or irregular	74 (5.9%)	63 (5.7%)	11 (7.7%)	
	Extra-thyroidal extension	31 (2.5%)	27 (2.4%)	4 (2.8%)	
**Echogenic foci** **(n, %)**	None or large comet-tail artifacts	1,006 (80.4%)	894 (80.5%)	112 (78.9%)	*0.965*
	Macrocalcifications	133 (10.6%)	117 (10.5%)	16 (11.3%)	
	Peripheral (rim) calcifications	35 (2.8%)	31 (2.8%)	4 (2.8%)	
	Punctate echogenic foci	78 (6.2%)	68 (6.1%)	10 (7.0%)	
**Internal structure** **(n, %)**	Homogeneous	586 (46.8%)	517 (46.6%)	69 (48.6%)	*0.651*
	Heterogeneous	666 (53.2%)	593 (53.4%)	73 (51.4%)	
**TI-RADS** **categories** **(n, %)**	TI-RADS 1	41 (3.3%)	35 (3.2%)	6 (4.2%)	N/A
	TI-RADS 2	11 (0.9%)	11 (1.0%)	0 (0.0%)	
	TI-RADS 3	214 (17.1%)	191 (17.2%)	23 (16.2%)	
	TI-RADS 4	822 (65.7%)	727 (65.5%)	95 (66.9%)	
	TI-RADS 5	164 (13.1%)	146 (13.2%)	18 (12.7%)	
**Localization** **(n, %)**	Right	665 (53.1%)	586 (52.8%)	79 (55.6%)	*0.804*
	Left	537 (42.9%)	479 (43.2%)	58 (40.8%)	
	İsthmus	50 (4.0%)	45 (4.1%)	5 (3.5%)	
**Number of nodules** **(n, %)**	Soliter	1,217 (97.2%)	1,078 (97.1%)	139 (97.9%)	*0.789*
	Multiple	35 (2.8%)	32 (2.9%)	3 (2.1%)	
**Parenchymal disease** **(n, %)**	Present	149 (11.9%)	132 (11.9%)	17 (12.0%)	*0.978*
	Absent	1,103 (88.1%)	978 (88.1%)	125 (88.0%)	

The distribution of nodules in the ND category is as follows: TI-RADS 1: 35 (3.2%), TI-RADS 2: 11 (1.0%), TI-RADS 3: 191 (17.2%), TI-RADS 4: 727 (65.5%), and TI-RADS 5: 146 (13.2%). In the AUS category, it is as follows: 6 (4.2%), 0 (0.0%), 23 (16.2%), 95 (66.9%), and 18 (12.7%), respectively. There are varying malignancy rates reported in the literature for the two groups.

Demographic characteristics, including gender and age, as well as the median nodule size, median TI-RADS score, and US features of nodules in the two groups, are presented in Table 1. Statistical analysis revealed no significant differences between these parameters in the two groups (*p* > 0.05).

There was no statistically significant difference in nodule composition between ND and AUS categories (*p* = 0.693). In the ND and AUS categories, the nodular composition feature exhibited similar and high rates of ‘Solid or almost completely solid’ (75.4% and 73.9%, respectively). Nodule echogenicity was found to have a high hyperechoic/isoechoic rate in both categories (63% and 70.4%, respectively). Additionally, nodule shape, margin, echogenic foci, distribution of TI-RADS categories, and other parameters showed similar distributions in both groups (Table 1).

## Discussion

In the specified date range of our study, 32% of the total thyroid FNA procedures performed (1218/3835) were ND, and 6% (242/3835) were AUS. The ND rate based on FNAC results varies significantly among studies in the literature [9–11]. Although ND rates of up to 47% have been reported, it is widely accepted to be in the range of 8% to 20% [12]. High ND rates may be associated with various factors, including advanced age, aspirin use, limited physician experience, and the absence of rapid on-site evaluation (ROSE), in addition to the sonographic characteristics of the nodule [13]. TBSRTC recommends that the AUS category should comprise less than 7% of all thyroid FNAC cases (3). This diagnosis exhibits significant variability, with an approximate reproducibility rate of only 50%, even among experienced pathologists. The frequency of AUS in various centers around the world varies widely, with reported rates ranging from 2.5% to 27% [6, 9, 14]. The variable rates observed in different centers can be attributed to factors such as the experience of pathologists and those conducting the procedure, as well as the utilization of ROSE.

In the ND category, out of 1,110 nodules, 37 (3.3%) were cystic, 8 (0.7%) spongiform, 228 (20.5%) mixed cystic and solid, and 837 (75.4%) solid. In the AUS category, among 142 nodules, 6 (4.2%) were cystic, 31 (21.8%) mixed cystic and solid, and 105 (73.9%) solid. In both categories, nodules predominantly displayed a solid composition. Some studies have found a statistically significant relationship between cystic nodules and ND [15, 16]. In our study, we did not find a statistically significant relationship between the ND category and nodule composition. The Bethesda Guidelines have subsequently revised their recommendations, suggesting that any sample with ample colloid should be regarded as benign, even in the absence of the six clusters of follicular cells. As widely known, the ACR TI-RADS guidelines do not recommend FNAC for the purpose of confirming or ruling out malignancy in TI-RADS 1 or 2 categories, except for therapeutic aspiration [8]. The number of nodules composing these categories in our study represents a small fraction of the population. Consequently, this may elucidate the absence of a statistically significant relationship between the composition of the nodules and the ND category. It is intuitively less probable for a cystic nodule with hemorrhagic content and/or an almost imperceptible wall to yield a diagnostic sample compared to a solid nodule.

Regarding the distribution of nodule composition, Salman et al. found that of 213 ND category nodules, 71 (33.3%) were mixed and 77 (36.2%) were solid. In the literature, solid composition is more commonly observed in ND nodules [17–19]. A high proportion of solid components is also generally reported in the AUS category [20, 21].

**Figure 4:**
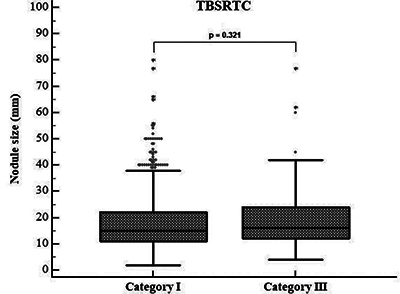
Relationship between nodule size and Bethesda Categories I (ND) and III (AUS)—distribution of nodule size in both categories. No statistically significant relationship was observed between nodule size and Bethesda categories I and III (*p* = 0.321). ND: Nondiagnostic, AUS: Atypia of Undetermined Significance, TBSRTC: The Bethesda System for Reporting Thyroid Cytopathology.

Literature questions the nodule size and cytological diagnosis link. Borges et al.’s study [22] found <1 cm nodules associated with ND, suggesting FNAC’s applicability regardless of size. Another study found no significant size–ND relationship [23]. In our study, ND and AUS showed no statistically significant size difference (<10 mm and ≥10 mm) (*p* = 0.475) (Table 1) (Figure 4). Thus, decisions on clinical management and follow-up should be independent of nodule size. However, according to the American Thyroid Association (ATA) guidelines, FNA is advised for nodules ≥1 cm with high or intermediate suspicion on US, ≥1.5 cm with low suspicion, and even ≥2 cm with very low suspicion. Sub-centimeter nodules don’t require FNA or cytological investigation [2].

In our study, ND nodules exhibited the following echogenicity characteristics: Anechoic 41 (3.7%), hyperechoic/isoechoic 699 (63.0%), and hypoechoic 370 (33.3%). For AUS nodules, the percentages were 6 (4.2%), 100 (70.4%), and 36 (25.4%). Notably, both categories showed a higher percentage of hyperechoic/isoechoic nodules. Asakly et al. [19] reported 70% hyperechoic/isoechoic nodules in Bethesda I, while Çetin et al. [18] found 23.1% in ND. Kulali et al. [17] observed 69.5% hypoechoic nodules in Bethesda I. Varying literature results highlight the need for extensive studies on US characteristics in these categories. There are numerous significant studies on the relationship between nodule echogenicity and malignancy [8]. Despite uncertainty in ND and AUS, the hyperechoic/isoechoic feature, suggesting lower malignancy, holds significance for both groups.

Per TBSRTC, ND carries a 13% malignancy risk, while AUS shows 22% [4]. Despite AUS’s higher risk, both groups often have nodules classified as TI-RADS 4, with a 5-20% cancer risk [8]. This suggests re-evaluating ND, often interpreted as benign. Considering TI-RADS scores, repeat FNAC is crucial. Persistent ND warrants excision. Previously, waiting 3 months before FNAC was advised, but ATA guidelines now permit immediate repeats [2].

Managing AUS nodules is challenging; the ATA advises considering sonographic appearance and patient preference for surveillance or surgical excision if FNA or molecular testing is inconclusive [2]. Studies using Bethesda criteria show surgery yields a 6% to 48% cancer probability for AUS nodules, averaging 16% [24]. In our study, most AUS nodules were TI-RADS 4, sharing a 4% to 20% malignancy risk with AUS nodules. Predicting FNAC outcomes requires considering US features and emphasizing their role in managing potential ND and AUS results.

Several limitations should be acknowledged in our study. First, the retrospective design of the study relies on existing medical records. Second, the study’s single-center nature may limit the generalizability of the findings to broader populations. Lastly, the sample size of the study may have limited the ability to detect smaller differences in US features.

## Conclusion

In summary, this study found no statistically significant differences between the ND and AUS categories regarding US characteristics. Also, there was no notable variance in nodule size distribution (<10 mm or ≥10 mm) between these categories. Managing nodules in these groups poses challenges due to diagnostic uncertainties and similarities in radiological appearances despite differing pathological results. Considering the potential for malignancy and diagnostic complexities, further investigations (e.g., molecular testing, surgery) may be necessary, and repeat FNAC should be contemplated.
